# Automatic Blastomere Recognition from a Single Embryo Image

**DOI:** 10.1155/2014/628312

**Published:** 2014-07-14

**Authors:** Yun Tian, Ya-bo Yin, Fu-qing Duan, Wei-zhou Wang, Wei Wang, Ming-quan Zhou

**Affiliations:** ^1^College of Information Science and Technology, BNU, Beijing 100875, China; ^2^Assisted Reproductive Medical Center, Navy General Hospital, Beijing 100048, China; ^3^Obstetrics and Gynecology Department, Navy General Hospital, Beijing 100048, China

## Abstract

The number of blastomeres of human day 3 embryos is one of the most important criteria for evaluating embryo viability. However, due to the transparency and overlap of blastomeres, it is a challenge to recognize blastomeres automatically using a single embryo image. This study proposes an approach based on least square curve fitting (LSCF) for automatic blastomere recognition from a single image. First, combining edge detection, deletion of multiple connected points, and dilation and erosion, an effective preprocessing method was designed to obtain part of blastomere edges that were singly connected. Next, an automatic recognition method for blastomeres was proposed using least square circle fitting. This algorithm was tested on 381 embryo microscopic images obtained from the eight-cell period, and the results were compared with those provided by experts. Embryos were recognized with a 0 error rate occupancy of 21.59%, and the ratio of embryos in which the false recognition number was less than or equal to 2 was 83.16%. This experiment demonstrated that our method could efficiently and rapidly recognize the number of blastomeres from a single embryo image without the need to reconstruct the three-dimensional model of the blastomeres first; this method is simple and efficient.

## 1. Introduction

Since the first IVF baby was born more than 30 years ago, IVF techniques have made considerable progress. However, the efficiency of IVF remains low. Thus, a great challenge that embryologists face is how to recognize the most viable embryo for implantation. Currently, doctors can only provide a rough evaluation for the viability of embryos by observing their morphological features using a microscope, which is subjective and is often dependent on the individual experience of doctors [1, 2]. In addition, this artificial method using visual observation is time-costly, and the method provides a qualitative and not a quantitative result. Computer-assisted automatic analysis of the embryo images can provide a more accurate quantification, which not only increases the objectivity to the process of selecting embryos but also accelerates recognition [3–6]. In general, in the day 3 embryo images, there are several important morphological indices for the evaluation of embryo viability: the number of blastomeres, uniformity of blastomeres, and symmetry [7–9]. In particular, the number of blastomeres on the third day after fertilization is a notably important feature. van Royen et al. [8] indicated that the day 3 embryos with good viability have the following features: (1) the number of blastomeres is more than 7; (2) the sizes of these blastomeres in one embryo are similar; (3) the fragmentation percentage is low. On the basis of these features, recognition of the number and relative size of blastomeres using a computer can track the timely development of blastomeres.

However, due to the transparency of blastomeres, overlap between blastomeres, and the ambiguity of the images themselves, it is difficult to automatically analyze blastomeres using a single embryo image. In addition, because embryos are three-dimensional, their images cannot well reflect the outlines, location, and other important information of blastomeres. To obtain the number, size, and location of blastomeres, Giusti et al. [10] reconstructed the 3D shape of the blastomeres from multiple images obtained in different focuses. However, reconstruction of the 3D model was relatively complicated and required a number of embryo images obtained in different focuses for each embryo. Pedersen et al. [11] proposed a 3D reconstruction algorithm, but it required manual segmentation of the 2D equator contours of the blastomeres. Ning et al. [12] recognized the cells by analyzing the embryo's video, which requires a long time. There are also other studies that helped to evaluate the quality of the embryos using the extracted multiple features, including morphological features and clinical features [13–16]. Recently, some researchers have employed the time-lapse images to assess the human embryos [17, 18]. Because our goal was to determine the number of blastomeres and not to precisely extract the contours, the above methods were complicated and required specific conditions. To the best of our knowledge, there has been no related research on the recognition of blastomeres by only using one embryo image. To this end, we aimed to automatically recognize the number of blastomeres from only the one day 3 embryo image after fertilization.

First, edge curves were obtained by effectively combining edge detectors, multiconnected point detectors, and some morphological operators; and the centers of the blastomeres were obtained. Finally, the number of blastomeres was obtained from an image using LSCF combined with two constraint conditions on blastomeres, including the upper limit of the blastomere size and the blastomere location. This proposed method only requires obtaining some edge curves and avoids the complicated 3D reconstruction of blastomeres.

## 2. Obtaining the Outlines of Blastomeres

The edges or part of the edges are very important for blastomere recognition. It is necessary to perform image preprocessing to obtain part of the edges before circle fitting. Every arc after preprocessing should be singly connected. With a large number of experiments, we developed a preprocessing method involving the following steps.

1. After filtering the gray image, the blastomere edges were obtained using the canny operator; however, the image may contain some noise and fragments, which should be deleted in the following steps.
2. Deletion of the multiconnected points and their domain, and the subsequent deletion of the formed short arcs that contain a small number of points. Multiconnected points refer to those that have more than three connecting directions. Only the single-connected arcs have a role for the fitting circle, such that the multiconnected points should be removed. In addition, the definition of a short arc is important because it will affect the fitting result. It is necessary to emphasize that multiconnected points should be recorded when they are deleted for the first time, such that they can be deleted for another time after performing the dilation thinning operation, which will make some turning points or multiconnected areas become smooth, and cannot be easily deleted by the detected turnings.
3. Deletion of the turning points of the single-connected arcs and recording.
4. Dilation thinning method. In the original image, a portion of edges were too wide to produce two nearly parallel arcs such that the dilation thinning method was necessary to reserve only one.
5. Deletion of new multiconnected areas again and those recorded previously. The same operation will be performed on the turning points.

**Figure 1:**
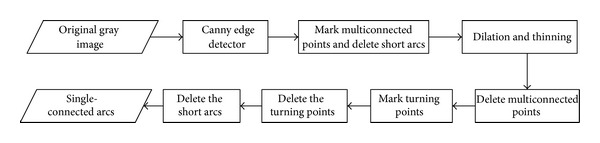
The flow chart of the preprocessing algorithm.

The flow chart of the preprocessing algorithm is shown in Figure 1. With the above processing, the arcs were ensured to be singly connected and well prepared for circle fitting in the next step.

## 3. Circle Fitting

**Figure 2:**
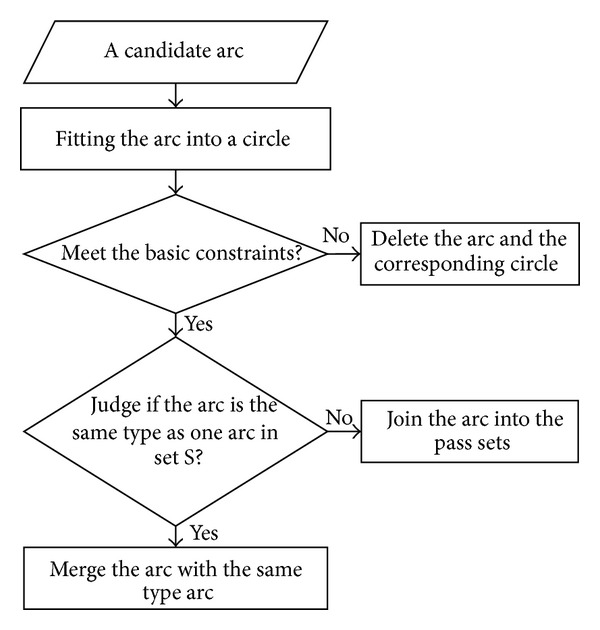
The process of the circle-fitting algorithm for blastomeres.

The shape of the blastomeres was round, such that it was reasonable to perform circle fitting for each blastomere. Due to the ambiguity of the blastomere edge, we could not ensure that there was a one-to-one correspondence between the fitting circle and the true blastomere because it was possible that more than one arc corresponded to the same circle, even though the fitting circle was nonexistent or wrong. Thus, some constraints were required to be established to exclude the unreasonable fitting circles. The flow chart for fitting could be observed in Figure 2. The main idea of blastomere recognition was to first remove the abnormal arcs via circle fitting with some constraints and then to judge whether the candidate arcs belonged to the same circle.

First, the whole fitting circle from the singly connected arc within the image according to the radius was assessed because the whole embryo usually completely appears in an image. If there are only parts of the circle that are in the image, we will delete the circle and remove the corresponding arc; otherwise, we will assess whether the arc belongs to the same type arcs, which correspond to the same circle. Let *S* denote the pass arc sets in which each arc corresponded to a different circle. If the arc is the same type as one arc in set *S*, it was merged with the same type arc; if not, then the new arc was added in the set *S*. The above manipulations were repeated until all of the candidates were confirmed. However, there may be some important issues, that is, how to determine whether two arcs belong to the same type. To address this problem, we developed three determinant conditions: (1) the centers of the two arcs of the same type should be near each other; (2) their radii should have a similar size; and (3) their corresponding sectors do not largely overlap.

### 3.1. Least Square Fitting

In this study, we employed the least square fitting method to fit the blastomere shape; with the optimization technique, we found the best matching function, which corresponded to a group of points by minimizing the square sum of error.

### 3.2. Removing the Abnormal Circles

**Figure 3:**
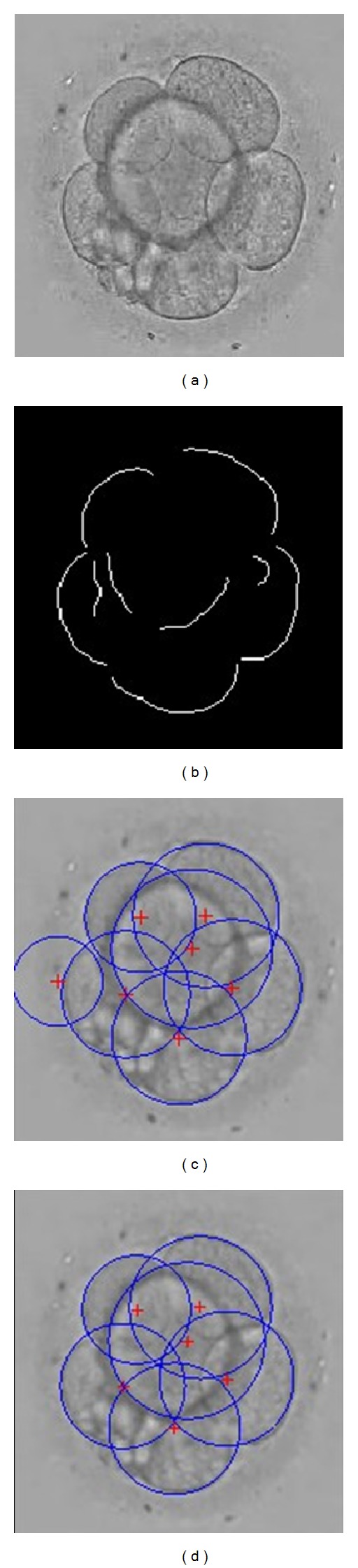
The recognition result of blastomeres under imposing the constraint.

After clipping and resizing the original image, embryos were usually located in the center of the image. To exclude the abnormal circle that was fitted wrongly, we introduced one constraint for the fitting circle. We examined whether the whole circle was within the image, which indicated that the circle should not be out of the range of the image. First, the distances between the center of the circle and the four edges of the image were determined. Next, these distances were compared with the radius of the circle. If the difference between any distance and the radius was less than a specific threshold, then the fitting circle was deleted. The recognition result with the constraint is shown in Figure 3. The original image is shown in Figure 3(a), and the singly connected arcs obtained are shown in Figure 3(b). The fitting results without and with the constraint, respectively, are shown in Figures 3(c) and 3(d). It was also observed that the leftmost circle in Figure 3(c) was removed according to the morphology and location features of the blastomeres.

### 3.3. Determination of “the Same Type of Arcs”

After preprocessing, we obtained many singly connected arcs and two or more arcs may come from the same blastomere edge. These arcs are known as “the same types of arcs.” To determine if two arcs are of the same type, the following steps are required. First, the distance between the two centers of circles confirmed whether it was sufficiently small, and then the two radii were inspected to determine whether they were similar. If these two cases were established, then the two sectors corresponding to two arcs were evaluated to determine any overlap. If there was no overlap, then the two arcs were defined as “the same type of arcs.” Thus, the corresponding sector of each arc was required to be indicated.

**Figure 4:**
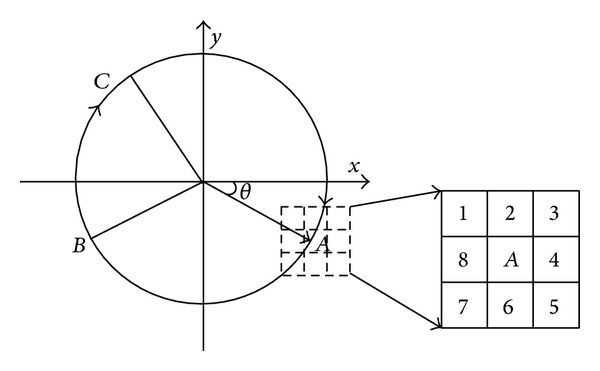
The representation of the arc in coordinates.

As shown in Figure 4, in the *x*, *y* coordinate system, the radius was rotated a period clockwise from the *x*-axis. Thus, any point *A* in the circle could be represented by an angle *θ*  (*θ* ∈ [0,2*π*)), and any arc *B* *C* could be uniquely represented as (*α*, *β*), where *α* corresponded to the starting point *B*, and *β* corresponded to the finishing point *C*.

For any arc, it was necessary to determine its starting point (clockwise), and this could be realized by inspecting the pixels in the local region centered at point *A*, as shown in Figure 4. If a point demonstrated only one neighbor pixel among their 8-neighborhood, it indicated the endpoints of the arc. Next, we determined the location of the quadrant of each endpoint. If it was located in the first quadrant, then we examined whether there were pixels in the label number 4, 5, or 6 in the 8-neighborhood. If these pixels existed, then this endpoint was the starting point; otherwise, it was the terminal point. Similarly, the starting point and endpoint could be determined in the other three quadrants. Next, the parameters *α* and *β* could be computed using the gradient of the two points. Finally, we determined whether the two sectors corresponding to two arcs overlapped by comparing the two angles *α* and *β*.

## 4. Experiments and Analysis

### 4.1. Parameter Setting

Preprocessing of the embryo image and the circle fitting involved some parameters. We performed experiments to select these parameters. In this study, we uniformly resized the image as 200 × 200 pixels, and the values of some important parameters were established by many studies. (1) When deleting the very short arcs after removing the multiconnected points for the first time, arcs whose pixels were less than 30 were removed. For the second time, the threshold was 20. For the last time deleting the short arcs, the threshold was 15. (2) When restricting the size of the candidates, the circles whose radii were between one-eighth of the row or column numbers of the original image, one-third of the radius of the fitting circle were reserved. (3) When eliminating the wrong circle candidates, apart from examining if two corresponding arcs are of the same type, it is also important to determine whether the entire fitting circle was within the image. Thus, a comparison of the distances between the center of the circle and the four edges of the image is required. In general, the circle is completely within the image, and we established the minimum distance between the center and the edges of the image as 2. If the value of the distance parameter was too large, then some circle candidates near the image edge may be missed; otherwise, some false circles with large radii may not be removed.

### 4.2. Experiments and Discussion

**Figure 5:**
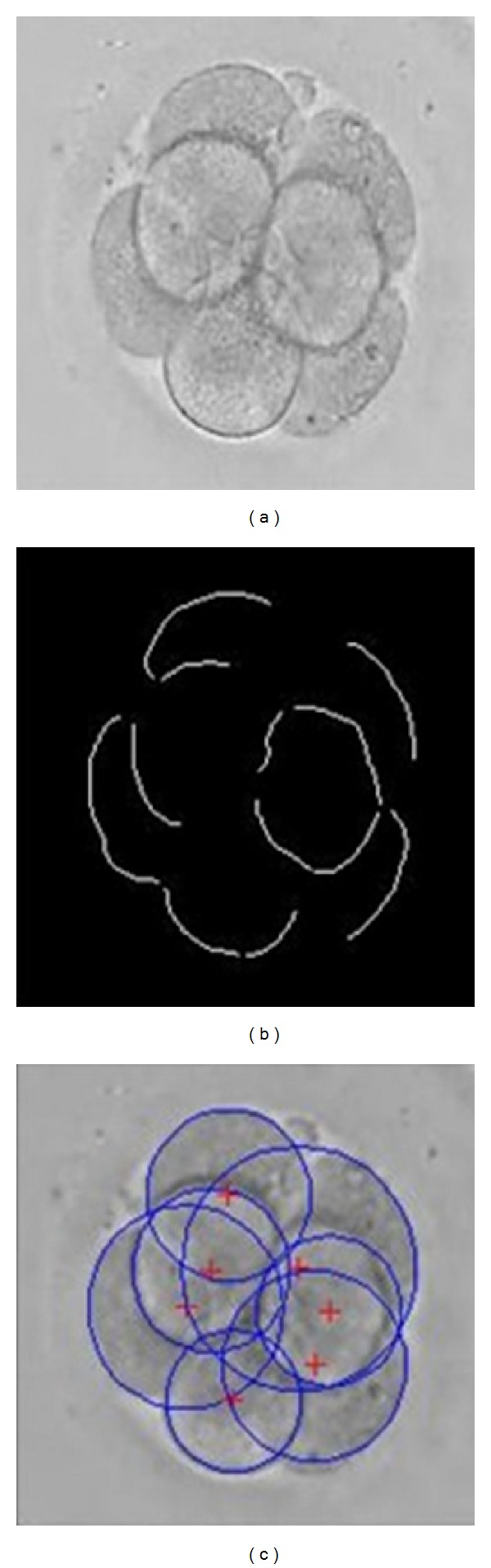
The result of the blastomere number identification. (a) Original gray embryo image; (b) preprocessing result; (c) number identification result.

We performed experiments on 381 embryo images from the Assisted Reproductive Medical Center of Navy General Hospital, PLA. Blastomere number identification results from one embryo image are shown in Figure 5. All blastomeres in the image were recognized using our method, and the recognition error in number was 0. These results showed that our method could accurately identify the number of blastomeres to some extent, although some fitting circles were larger than the corresponding real blastomeres. The reason may be that the blastomere shape was not truly round. However, this did not affect the identification number and the relative location of the blastomeres.

**Figure 6:**
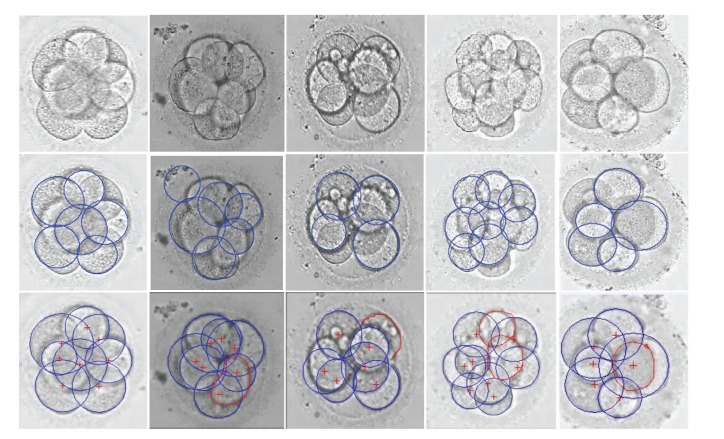
The blastomere recognition results of the different methods. Row 1 shows original embryo images; Rows 2 and 3 show the recognition results of the Hough transform-based method and our method, respectively. The fitting results of the different methods are shown in each column.

We compared our method with a classical circle detection method based on Hough transform [19], that is, Hough transform-based method.  Figure 6 shows the detection results of the different methods. The first row shows 5 embryo images randomly chosen from 382 images. The second row shows the recognition results of the Hough transform-based method, and the third row shows the results of our method. In each column, the original image, the recognition results from the different methods are shown from top to bottom. The fitting circles were marked in blue, and the edges of the undetected blastomeres were manually depicted in red.

We regarded the assessment of the embryologists as the gold standard, and the number of blastomeres in the images from left to right was 8, 7, 8, 10, and 7, respectively. Corresponding to these original images, the recognition results of the Hough transform-based method were 6, 6, 4, 8, and 5, respectively, and the error numbers were 2, 1, 4, 2, and 2, respectively. As can be seen the Hough transform-based method usually missed some blastomeres, and sometimes this is very serious, for example, as in the fourth image. As for the proposed method, the recognition results were 8, 6, 7, 8, and 6, respectively, and the error numbers were 0, 1, 1, 2, and 1, respectively. For the first image, the blastomeres were equal in size and symmetrical. Although the two blastomeres in the middle were slightly vague, our method could reserve part of their edges and finally provided the correct circle. For the second image, only one blastomere was not recognized and this might be due to the overlap between blastomeres and impurities; the edges of the undetected blastomere were deleted by mistake in the preprocessing stage. For the third image, due to the existence of fragmentation, the blastomere edges in the upper right corner were removed with the fragments near the blastomere. Thus, this blastomere was omitted. For the fourth image, the development of this embryo was rapid such that the number of blastomeres was relatively large. There were two blastomeres missing and thus could not be detected. The reason was that the boundary of one blastomere was not very sharp for detection even in the first process of edge detection, and the boundary of the other blastomere was cut into very short arcs by the neighboring blastomeres. For the last image, due to the cover of a large blastomere, a small blastomere could not be recognized.

A comparison of our automatic algorithm with the Hough transform-based method on all embryo images was performed.  Table 1 lists the recognition results.

From Table 1, the Hough transform-based method had a low recognition rate for the blastomere recognition, and the false recognition number more than 2 was 66.09%. The reason is that the Hough transform-based method is susceptible to the influence of the object illumination and intensity inhomogeneity of images, and it would be impossible to detect circles in complex images. For our method, there were 82 images in which the blastomeres were all recognized and the number of false detection was 0, occupying 21.59%; 146 images with 1 blastomere undetected, occupying 38.27%; and 89 embryo images with 2 blastomeres undetected, occupying 23.30%. This indicated that images with equal to or less than 2 blastomeres incorrectly recognized accounted for 83.16%. It can be seen from Table 1 that the recognition rate of our model is higher than that of the Hough transform-based method as a whole. However, the proposed method still fails to recognize a small number of blastomeres, which was mainly caused by a false-negative judgment. The relatively large differences in the details between embryo images resulted in its difficulty in some parameters in the preprocessing to adapt to all images. In addition, our method was based on a single 2D embryo image to recognize blastomeres, and this 2D image was not able to fully reflect the 3D information of embryos. However, this method could still identify the vast majority of blastomeres. Furthermore, it exhibits a low complexity, small amounts of computation, and a fast recognition speed. In particular, this method showed good recognition performance in the embryo images in which the boundary was relatively clear. Thus, this method is very meaningful and helpful in blastomere recognition, which can assist in human labor, due to the percentage of embryo images with less than 2 undetected blastomeres in 8-cell stage.

## 5. Conclusions

The number of blastomeres on day 3 is a very important morphological index for evaluating the quality of an embryo. Currently, the embryologists' subjective observation is usually the deciding factor. Related studies on the automatic recognition of the number of blastomeres using an embryo image have been limited. In this study, we proposed an automatic method for the recognition of the number of blastomeres on day 3 based on LSCF, which does not first require the reconstruction of the 3D model of blastomeres. In addition, it can efficiently recognize the blastomeres using a single embryo image. Images with no undetected blastomeres occupied 21.59%, and images with less than 2 undetected blastomeres occupied 81.36%, which meets the clinical requirement. If our method is combined with images obtained in different focuses, then the recognition accuracy is expected to be greatly enhanced.

## Figures and Tables

**Table 1:** The blastomere recognition rate (%) of the different methods.

	Numbers of blastomeres recognized incorrectly			
	0	1	2	>2
Hough transform-based method	6.30	10.88	16.73	66.09
Proposed method	21.59	38.27	23.30	16.84
